# Instructions to a Tyro on the Use and Application of the Forceps in Midwifery*


**Published:** 1820-09

**Authors:** 


FOR THE LONDON MEDICAL AND PHYSICAL JOURNAL.

### *Instructions to a Tyro on the Use and Application of the Forceps in Midwifery.**

THE cases to which the forceps are applicable, are all those in which there is a combination of the following circumstances: 1st, the safety of the woman must require delivery by art; 2d, the head must present; 3d, the labour must be sufficiently advanced; 4th, the dimensions of the pelvis must be sufficient to admit the passage of the head, without such a reduction of its bulk as is produced by an evacuation of its contents.

* The author of this paper has chosen that it should appear without his proper signature; but he is known to us; and we assure the reader, that he is a man, of very extensive experience in the practice of medicine, whose observations and opinions merit the deepest attention. Thus much it is right to state, in order to obviate the prejudice with which an anonymous paper is commonly regarded. We express no judgment on the value of this article; but would persuade the reader to peruse it with a disposition of mind proper for the discovery of its merit.—EDIT.

1. Delivery by art becomes necessary when the natural powers are inadequate to this end. The natural powers may be unequal to accomplish delivery, either because they are inordinately weak, or because the resistance, arising out of the relative size of the head and the diameter of the pelvis, is too great. The first of these cases, or that in which delivery cannot be accomplished from defect of pains, is termed a case of arrest, and may be thus exemplified. A woman felt slight pains on Monday evening about six o'clock, and the membranes ruptured soon after: she was kept awake by these pains during the night, and similar pains continued the whole of Tuesday. Upon examination twenty-four hours from their commencement, the os uteri was dilated to the size of a shilling; there was some mucus in the vagina; and, the finger being introduced between the os uteri and the head of the child at the time of a pain, the pressure was found to be very trifling. On Wednesday morning at six o'clock, the same kind of pains were reported to have continued during the night, and the os uteri was nearly half dilated. Similar pains, with no other variety than that of succeeding each other sometimes faster and sometimes slower, continued till the evening; when, at seven o'clock, the os uteri was fully dilated, and the head between two and three inches from the os externum. At eight o'clock on Thursday morning, the head was within three-quarters of an inch of the os externum. At this time the woman appeared much fatigued; she had had but little sleep, and the pains, which were always short and weak, had rather abated within the last four or five hours. At six o'clock the same day, exhaustion was still more apparent: the pains were weaker, and succeeded each other at long intervals, and the head almost pressed upon the os externum. That part of the labour had not arrived which required the strongest expulsive efforts: whether, under such circumstances, these efforts are to be waited for, is the chief point of practical discrimination. The patient may sleep in this stage, and adequate pains might afterwards come on; or the state of exhaustion might proceed rapidly from this stage; or the soft parts might sustain mischief, might suppurate, or finally slough; or the introduction of the catheter, if necessary, may be difficult, perhaps impracticable,—by deciding, in this stage of the labour, to trust the result wholly to the natural powers. As there can be no certain rule by which we can pronounce that adequate pains will, or will not, come on, it is right to adopt the practice which involves the least risk. In the case just described, the pulse was quick, the tongue white, and the woman much fatigued: the practice which risked the least was supposed to be to deliver, without waiting for pains which might never come on. She was accordingly delivered with great facility by the forceps, three days from the commencement of labour, and recovered as favourably as if the delivery had been natural under the best circumstances.

But a labour somewhat similar to the one described might require a different treatment. The pains might be violent, but useless, in the commencement of labour; opiates may not abate their violence; and, when more efficient pains come on, these also may be of a kind which exhaust the woman rapidly, without a corresponding progress of the labour. By the time the head is brought near the os externum, which may be in twenty-four hours from the commencement of labour, the woman's powers may be unequal to any further effort. No time is gone by, and we may wait a few hours; but, other symptoms of exhaustion continuing, and the pains not recurring, it will in this case be proper to deliver without any great loss of time. Can we then deduce any general rule for the time when the use of the forceps becomes proper in cases of arrest? In reply to this question, perhaps the following rule might be hazarded, viz. in no case to wait longer than twelve hours after delivery by the forceps with facility becomes practicable; and, in some cases, where the labour has been long protracted, or there is from any other cause reason to think that the natural powers of the woman might speedily fail; in such cases, to deliver with the forceps as soon as delivery might be accomplished with safety.

The necessity of delivery from preternatural resistance, may be exemplified in the following case. A woman was taken in labour at eight o'clock on Sunday evening: the pains were strong; the os uteri soon began to dilate; the membranes ruptured at twelve; the pains continued, always strong, but with some variety in the intervals of succession, until six o'clock Monday morning, when the scalp, which had formed a tumor at the time of a pain, dilated the os externum. The strength of the pains diminished for the next two hours, and the woman appeared much fatigued: for the next two hours the pains were strong again; the head, however, did not advance; the pains, for the next three hours, were sometimes quick, sometimes distant from each other, always weak and ineffectual, and the woman was apparently incapable of any further efforts. At this time, (seventeen hours from the commencement of labour,) she was delivered with forceps; and the necessity for delivery in this case appeared quite equal to that in the case of arrest in which labour of a different description had continued three days. It is difficult, perhaps impossible, to define a rule with respect to the period at which it is proper to deliver by art, which will agree with every variety of case. The only one which can be proposed, and which must of necessity be somewhat vague and indefinite, is to deliver with the forceps, as soon as it can be done with facility and safety, in every case in which there is reason to believe that the life of the mother might be endangered by further delay. There is, under proper circumstances and judicious management, no danger in the employment of forceps: if there is any danger from delay, there can be no doubt about the line of practice to be adopted; if there were some danger in either alternative, the decision would then be governed by a comparison of *the degrees of danger;* but, if there *may* be danger in one, and can be none in the other, it is unnecessary to say, that the practice by which nothing is risked should be unhesitatingly preferred.

To proceed with the other circumstances which must concur to render the employment of the forceps practicable.

2. The head must present. The head may present either with the face towards the pubes, or towards the sacrum, of the mother: the latter is the natural presentation. It is directed, in some systems, to change the former for the latter position in the earlier stage of labour: this may sometimes be done; but such a deviation from the strictly-natural presentation is rarely of any great consequence, except where there is some distortion of the pelvis. The importance of the circumstance in this case may be so great, that a distortion which, in a first labour, has admitted delivery by the forceps when the face was towards the sacrum, in a second labour has required the head to be opened, because the face was towards the pubes. This has happened in my own practice; but, except in a distorted pelvis, or in some other cases of difficult labour without positive distortion, where the most trifling increase of bulk, as well as the least deviation from the best position, are of importance, it matters very little whether the face of the child opposes the sacrum or the abdomen of the mother; and, in these cases, the impediment by which the labour is rendered unusually difficult, will probably also render a change in the position of the head difficult, if not impracticable. Whatever the position of the head might be, the forceps are applicable under every variety in this respect.

3. The labour must be sufficiently advanced. It is commonly said, the forceps may be applied when an ear of the child may be felt from the os externum. This is a good general rule; but the limitation is on some occasions rather too strict, and it is otherwise not unobjectionable. One who is not a proficient in the art, or who is a learner, or whose tact is not matured, may not be able to discover an ear, or be certain that he feels the ear, when delivery with the forceps may be perfectly easy. It may be said, in general, that a labour is sufficiently advanced to admit the use of the forceps, when the os uteri is so fully dilated that the circle of it is quite, or almost, obliterated, and when the head is within half an inch of the os externum. A very trifling difference in the descent of the head makes a considerable difference to a beginner in the facility of using the forceps: if the head, at the time of introducing the forceps, is an inch, or an inch and a half, from the os externum, the lock will perhaps be, when the blades are introduced, within the labia, and this circumstance will cause some embarrassment: the soft parts will be pinched; the blades may be a little withdrawn; then one of them slips, and, in attempting to replace it, the other slips, or may be turned quite around, so that both blades may embrace one side of the head. Such accidents cannot occur to one who is master of the instruments, but they may foil the attempts of a tyro; and therefore it is better, until some facility is acquired by practice, not to attempt delivery with the forceps, except under circumstances of great urgency, until the head almost presses upon the os externum. But these restrictions do not apply to one who is familiar in the use of the forceps. It is possible to deliver safely with these instruments when the os uteri is not more than half dilated, and when the head is three inches from the os externum; and this, too, although the impediment to natural delivery should be occasioned by malformation of the pelvis. Those who are so far masters of the forceps as to be able to deliver safely under such circumstances, (and he who cannot is by no means a well-qualified practitioner,) will often preserve both the mother and the child, where others, less skilful in the use of the forceps, would feel no doubts about the necessity of opening the head.

4. The pelvis must be of sufficient dimensions to admit the passage of the head without an evacuation of its contents. The success of the trial of the forceps in cases of distorted pelvis, will, of course, depend upon the degree of its malformation: in the worst cases of this description, where the passage of the entire head is mechanically impossible, the forceps are, of course, totally inapplicable; but, where it is calculated that the aperture of the pelvis, although contracted, is equal to a space of from two inches and a half to three inches, I believe delivery by the forceps to be frequently practicable. The forceps which are adapted for delivery in cases of this description, will require that the blades should be from eight to nine inches in length, certainly not less than eight inches, and not more than nine. With respect to the shape of the forceps, this is a matter of choice, in which practitioners are not agreed: I prefer perfectly-straight blades, because more simple than the curved ones both in their construction and use. The practitioner has not, with straight forceps, to perplex himself with an upper and a lower blade; it matters not which blade is introduced above, or which below, and all the advantages, if any, of the curved forceps may, with the straight ones, be obtained by a common attention to the axis of the pelvis. Although the forceps may frequently succeed in very difficult and unfavourable cases, in the hands of one who is familiar in their use, the persevering trial of them in cases of the above description would amount to a culpable temerity in one whose practical knowledge of their application is incomplete.

In those cases of contracted pelvis in which the forceps are successful, they do not act by compressing the head; for the diameter of the forceps from blade to blade will always be more than three inches; but the force of extraction compels the bones of the head to yield wherever the resistance is the greatest.

*Application of the Forceps, &c.**—1. Let the woman be placed on her left side, with the shoulder and head a little raised, and her hips brought completely over the edge of the bed. The best position for the operator is kneeling, so as to face the axis of the pelvis.

2. The first stage of the operation, is the introduction of the blades. If an ear can be felt, this will be a guide for their direction; if an ear cannot be felt, the first or superior blade should be introduced, not immediately under the arch of the pubes, nor quite laterally, but between these directions, so that the concave surface of the upper blade faces the tuber ischii of the opposite side: the direction of the second or inferior blade, is to be such that its concave surface exactly opposes that of the first.

3. The fore-finger of the left hand is to be passed as high as it can reach, at all events within the circle, if any remains, of the os uteri; and the blade of the forceps, at the time of its entering the uterus, is to be pressed forward with the right hand, between the head of the child and the fore-finger of the left hand, which thus protects the os uteri from injury: further than this, the blade is to be pressed forward steadily, inclining the pressure of its extremity rather upon the head of the child than against the uterus, until it appears to embrace the head, or until the lock is arrived at, or near the os externum. One blade being thus introduced, it is to be held firmly by the nurse or assistant, and the handle raised slightly against the side on which it is introduced, which will give room for a more easy introduction of the second blade. Both blades being introduced by a similar proceeding, each handle is taken hold of firmly with either hand: their length should be equal; it should

* If any apology is necessary for giving in this place instructions on a department which is, in general, so ably taught, the writer has only to say, that, in the earlier periods of his practice, such instructions would have been acceptable to him; and, for the same reason, might be useful to others.

be ascertained that the lock is clear of the labia, &c.; and, if either blade is introduced too far, it should be a little withdrawn. If, after the blades are introduced, they are held loosely, the actions of the uterus will frequently so completely displace them, as to render a fresh introduction necessary. Being once properly introduced, their position must be maintained by holding, or moving, them with a sufficient degree of firmness. It being ascertained that the blades are properly introduced, that they embrace the head, that the concave surfaces oppose each other, that the handles are of equal length, and that the soft parts are not in the way, the instruments are now ready to be locked; which is the second stage of the operation, and is thus performed:

4. The handle of each blade is grasped firmly in either hand; the handles are then made to cross each other, on the side of the stop, or shoulder, which forms the lock. The crossing of the handles must be continued until the blades cross each other; the handles are then pressed together, and the instrument must thus infallibly be locked. It may appear to a novice that the blades are locked, when, in fact, the shoulder of one blade is only within that of the other. Under this circumstance, any attempt at extraction must necessarily fail; and it is obviated only by continuing to cross the handles until the blades cross each other, which must, of course, be done on the side of the stops. When the instruments are fairly locked, it is ascertained by feeling the stop or shoulder of each blade on the outside of the other blade, which cannot be done in any other relative state of the blades. The application of the forceps is now completed; and, in order to preserve the place and relation of the blades, it is necessary to press the handles together, with no very great force, and secure them by means of a napkin, ribband, or tape.

5. The third stage of the operation, is the extraction of the child, which should be attempted at the time of a pain, and the force employed must be in proportion to the resistance to be overcome. If there are no pains, the extraction should be made (in the direction of the axis of the pelvis when the head is high up, and afterwards in the axis of the vagina,) at intervals; relaxing a little, between each attempt, the pressure of the instruments upon the head of the child. At the time of extraction, it is essential to bear in mind that the pressure of the instruments upon the head must be proportionate to the force necessary to be employed in the extraction. The hands have thus a double duty to perform at the same time; one, to compress the handles with so much firmness as to obviate the possibility of the slipping, and consequently reiterated introduction, of the blades; and the other to employ so much force in the extraction as is necessary to accomplish the delivery. In difficult cases, this double duty is no joke; it is very hard work, and the hands are apt to feel it for some days; but a want of attention to both these objects at the same time may admit the repeated slipping of the blades, and eventually a failure, in cases where success is otherwise possible. The principal resistance being overcome, very little force is necessary to bring the head of the child through the os externum. In this part of the operation, when the os externum begins to be dilated by the pressure or proximity of the head, the perineum is to be supported by an assistant; and this may be done in two ways, one in which it will be useful, and one in which it will be useless. If the assistant makes a vague and diffuse pressure upon the perineum, this part might be lacerated as readily as if no such pressure were made: the point at which the perineum chiefly requires support, is exactly at the inferior junction of the labia. Any woman who happens to be present at a labour is competent, if properly directed, to do any thing which is required of an assistant, such as to hold the first blade of the forceps while the operator is introducing the second, support the perineum, &c. L. G.

*Bath; June* 1820.

P.S. It is by no means the object of this paper to supersede the excellent instructions which are delivered elsewhere, both in lectures and in books. The design of the writer was to give some abstract rules, principally with respect to the locking of the forceps, the want of which, in the earlier part of his practice, he himself felt; which laid him under the necessity of studying the mechanism of the instruments, in order to acquire them. Although it frequently happens, when the blades of the forceps are properly introduced, that they lock almost without an effort on the part of the operator, yet it does also frequently happen that the handles, when introduced, either do not cross at all, or else cross each other at the sides opposite to the stops, or shoulders, and, consequently, the instruments cannot lock until they are adjusted according to the rule delivered, or until this latter relation is reversed. It is conjectured, also, that it is better to practise invariably upon a rule which must be infallible, than to trust in any degree to chance.

